# Internal poly(A) replacement strategy within 3′ UTR for live-attenuated tick-borne encephalitis virus

**DOI:** 10.1128/jvi.00781-26

**Published:** 2026-09-16

**Authors:** Qiu-Yan Zhang, Zhe-Rui Zhang, Cheng-Lin Deng, Jing Wang, Hong-Qing Zhang, Han-Qing Ye, Ya-Nan Zhang, Bo Zhang

**Affiliations:** 1 Chinese Academy of Sciences, State Key Laboratory of Virology and Biosafety, Wuhan Institute of Virology 74614, Wuhan, China; 2 Hubei Jiangxia Laboratory 854985 , Wuhan, China; University of North Carolina at Chapel Hill, Chapel Hill, North Carolina, USA

**Keywords:** tick-borne encephalitis virus, 3ʹ UTR, poly(A) replacement, Δ1-642/poly(A), live-attenuated vaccine

## Abstract

Tick-borne encephalitis virus (TBEV), a prominent orthoflavivirus transmitted by ticks, poses a risk of potentially fatal encephalitis in humans. Currently, the only available inactivated vaccines have limited usage, underscoring the need for developing novel vaccine strategies. The 3′ untranslated region (UTR) of TBEV can be divided into variable and conserved regions, where structured RNA elements play crucial roles in viral replication and virulence. In this study, we found that deleting sequences in both the variable region and most of the conserved region within the 3′UTR eliminated viral replication. However, inserting a poly(A) tract effectively restored viral propagation. Notably, the recovered virus variants, Δ1-642/poly(A), exhibited high attenuation in mice. A single-dose immunization with Δ1-642/poly(A) induced robust TBEV-specific IgG, neutralizing antibody, and T-cell immune responses, providing complete protection against lethal challenges with wild-type virus. Collectively, our findings demonstrate that poly(A) replacement represents a viable strategy for developing live-attenuated TBEV vaccines.

**IMPORTANCE:**

Orthoflaviviruses, encompassing mosquito-borne dengue virus (DENV), yellow fever virus (YFV), Zika virus (ZIKV), West Nile virus (WNV), and tick-borne encephalitis virus (TBEV), represent a substantial global public health and economic burden. The 3′ UTR is critical for viral replication and pathogenesis, as exemplified by the ongoing clinical development of the tetravalent Butantan dengue vaccine (with deletion in 3′ UTR). Our previous study on WNV-poly(A) variants proposed a novel live-attenuated vaccine design via 3′ UTR engineering, yet its broad applicability remained unclear due to extensive 3′ UTR heterogeneity. In this study, we successfully rescued a TBEV poly(A) virus using an analogous strategy. This virus was highly attenuated in mice, and a single immunization provided long-term complete protection against lethal wild-type virus challenge. Collectively, our data validate the poly(A) replacement approach as a potentially universal platform for developing live-attenuated vaccines against both mosquito-borne and tick-borne orthoflaviviruses.

## INTRODUCTION

Tick-borne encephalitis virus (TBEV), a member of the genus *Orthoflavivirus* in the family *Flaviviridae*, is a major arthropod-borne virus responsible for severe and potentially fatal encephalitis in humans. Each year, thousands of severe neurological cases are reported in endemic regions across Europe and Asia, including northern China (1, 2). Currently, no licensed antiviral therapies are available for TBEV infection, and the existing inactivated vaccines exhibit limited application and require multiple-dose immunization regimens (3, 4). Live-attenuated vaccines (LAVs), such as approved Dengvaxia (CYD-TDV), TAK003, and BuTantan for dengue virus, YFV 17D, and JEV SA14-14-2, mimic natural infection and can elicit long-lasting humoral and cellular immune responses following a single immunization, representing a highly promising approach. Thus, the development of novel strategies for TBEV live-attenuated vaccines is urgently needed.

The orthoflavivirus genome is a ~11 kb, single-stranded positive-sense RNA with a type I cap at the 5′ end and no poly(A) tail at the 3′ end. Its single open reading frame (ORF) is flanked by highly structured 5′ and 3′ untranslated regions (UTRs). Determinants of virulence are located in both the coding region and UTRs, particularly the 3ʹ UTR (5–7). The 3ʹ UTR of orthoflavivirus varies in length and contains multiple functional RNA elements. Comparative alignment reveals that except for the highly conserved small hairpin 3ʹ-stem-loop (sHP-3ʹSL) structure at the 3ʹ terminus, the upstream elements show great variability that may vary depending on virus species. For instance, mosquito-borne orthoflaviviruses (MBFV) feature two characteristic stem-loop (SL) and dumbbell (DB) structures, whereas tick-borne orthoflavivirus (TBFV) represented by TBEV lacks DB structures but possesses two distinct Y-SL or GC-SL structures (Fig. S1). These structured RNA elements are vital for viral replication, transmission, and virulence (6, 8–10). Increasing studies have indicated that mutations or/and deletions of the SL or DB structure attenuate the virulence of various MBFV in mouse models, such as JEV, DENV, WNV, and ZIKV (5, 6, 11–14), highlighting the potential of the 3′ UTR as a target for orthoflavivirus vaccine design (14–16).

In our previous study, we have developed a live-attenuated vaccine (LAV) candidate, WNV-poly(A), by replacing the 5′ portion (corresponding to SL and DB domains in WNV) of the 3′ UTR with an internal poly(A) tract, which elicited robust and long-lasting immune responses, conferring full protection against the wild-type (WT) WNV challenge (17). In the case of TBEV, the presence of internal poly(A) tracts within the 3ʹ UTR has been documented (18, 19). These tracts, along with the variable region in the 3ʹ UTR, are considered crucial determinants of TBEV virulence (15, 20). Mouse studies showed that, compared to the TBEV Hypr strain without a poly(A) tract, the Torö−2003 strain with 38A exhibited a significantly higher level of virulence (18). Furthermore, extensive investigation of the quasispecies populations revealed that no specific length of the poly(A) tract was preferred during virus replication in cell culture or in the brains of infected mice (18). Given this, the applicability of the internal poly(A) replacement strategy employed for WNV attenuation to TBEV remains uncertain.

In this study, we evaluated the feasibility of the poly(A) replacement strategy for the development of TBEV vaccine. Initially, we identified that the deletion of the entire variable and majority of the core region in the 3′ UTR (Δ1-592 and Δ1-642) failed to produce viruses. However, with the insertion of poly(A) sequences, the resultant Δ1-592/poly(A) and Δ1-642/poly(A) variants could efficiently replicate in cell culture. Notably, compared with WT viruses, the Δ1-592/poly(A) showed no significant reduction in mouse mortality, whereas the Δ1-642/poly(A) variant exhibited marked attenuation. Moreover, a single-dose immunization with the Δ1-642/poly(A) variant was sufficient to elicit robust, long-lasting neutralizing antibody and cellular immune responses, thereby conferring full protection to immunized mice against lethal challenge of WT virus. In summary, our findings suggest that the internal poly(A) replacement strategy is feasible for TBEV, further supporting the notion that this strategy could serve as a universal platform for the development of live-attenuated orthoflavivirus vaccines.

## RESULTS

### Large sequence deletion in the 3′ UTR was lethal for TBEV

**Fig 1:**
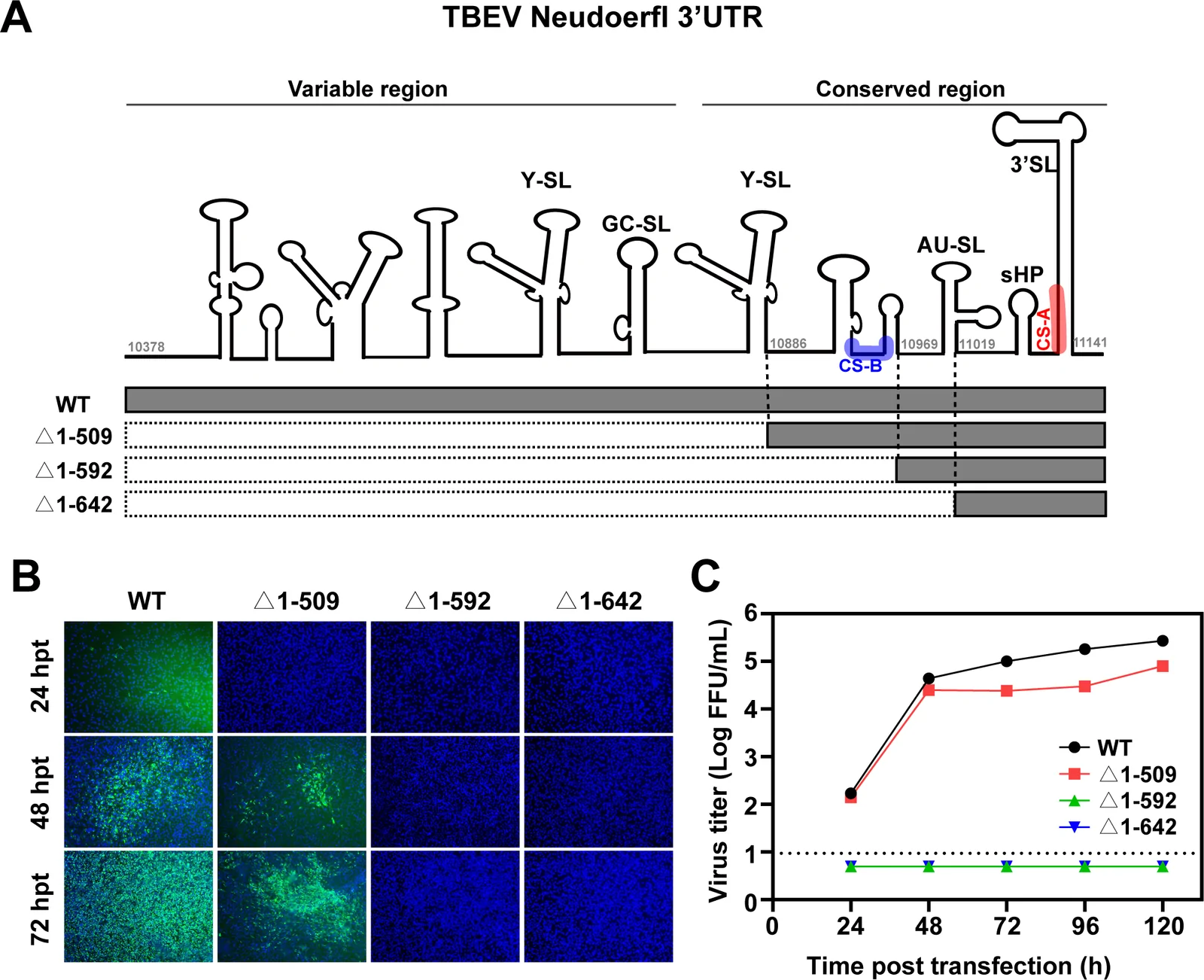
Engineering and characterization of deletion mutants. (**A**) Secondary structure schematic of TBEV Neudoerfl 3′ UTR. TBEV 3′ UTR was divided into variable and conserved regions. The position of cyclization sequence CS-A and CS-B was marked in red or blue, respectively. The deleted sequences were shown with dotted boxes. (**B**) One microgram of wild-type (WT) or different-sequence deletion mutant RNAs (Δ1-509, Δ1-592 and Δ1-642) were transfected into BHK-21 cells. Transfected cells were fixed at 24, 48, and 72 h post-transfection (hpt), and the expression of E protein at the indicated time points was detected by indirect immunofluorescence assay (IFA). The 4G2 monoclonal antibody and FITC-conjugated goat anti-mouse IgG (dilution in 1:125 with PBS) were used as primary and secondary antibodies, respectively. (**C**) Viral growth curves of WT, Δ1-509, Δ1-592, and Δ1-642 mutant viruses in BHK-21 cells. The supernatants from RNA-transfected cells at different time points were collected and subjected to focus-forming assay to determine the virus titers.

The 3′ UTR of TBEV was divided into two distinct domains, the 5′ variable and 3′ conserved regions. The conserved region contains sequences necessary for viral genome replication, such as the cyclization sequence (Fig. 1A) (19, 20). Previous studies have shown that deletion of the entire variable region in the 3′ UTR of the Neudoerfl strain did not affect viral replication in cell culture (19). Moreover, the 3′ UTR of TBEV also contains adenylate-rich (A-r) segments with significantly higher adenosine ribonucleotide composition, like WNV (17), which reminded us that large segment deletion and replacement of poly(A) in the 3′ UTR may also serve as a replication-effective and attenuated strategy for TBEV. For WNV-poly(A), the poly(A) tract replaced all the replication enhancer elements within the variable and part of the conserved domain of the 3′ UTR, which was indispensable for virus replication and virulence.

To determine the optimal poly(A) replacement strategy of TBEV, we first assessed the impact of deleting longer sequences containing all the variable region and partial conserved region on viral replication, such as Δ1-509, Δ1-592, and Δ1-642. The deletion mutant RNAs were transfected into BHK-21 cells, and virus production was identified by indirect immunofluorescence assay (IFA). As shown in Fig. 1B, the number of E-positive cells for mutant Δ1-509 was slightly decreased compared to WT, consistent with previous report (19), but no positive cells were observed for mutants Δ1-592 or Δ1-642. Moreover, to further compare the viral replication efficiency, the growth kinetics of the mutant viruses in BHK-21 cells was determined by quantifying viral loads at different time points post-transfection (Fig. 1C). Consistent with the IFA results, the Δ1-509 mutant replicated slightly slower than that of WT virus at all time points, and no foci-forming units were detected in Δ1-592 or Δ1-642 mutants, indicating that these mutations were lethal for virus production. These two lethal mutations could serve as potential sites for inserting poly(A) tract to generate viable viruses.

### Engineering and characterization of recombinant TBEV poly(A) replacement

**Fig 2:**
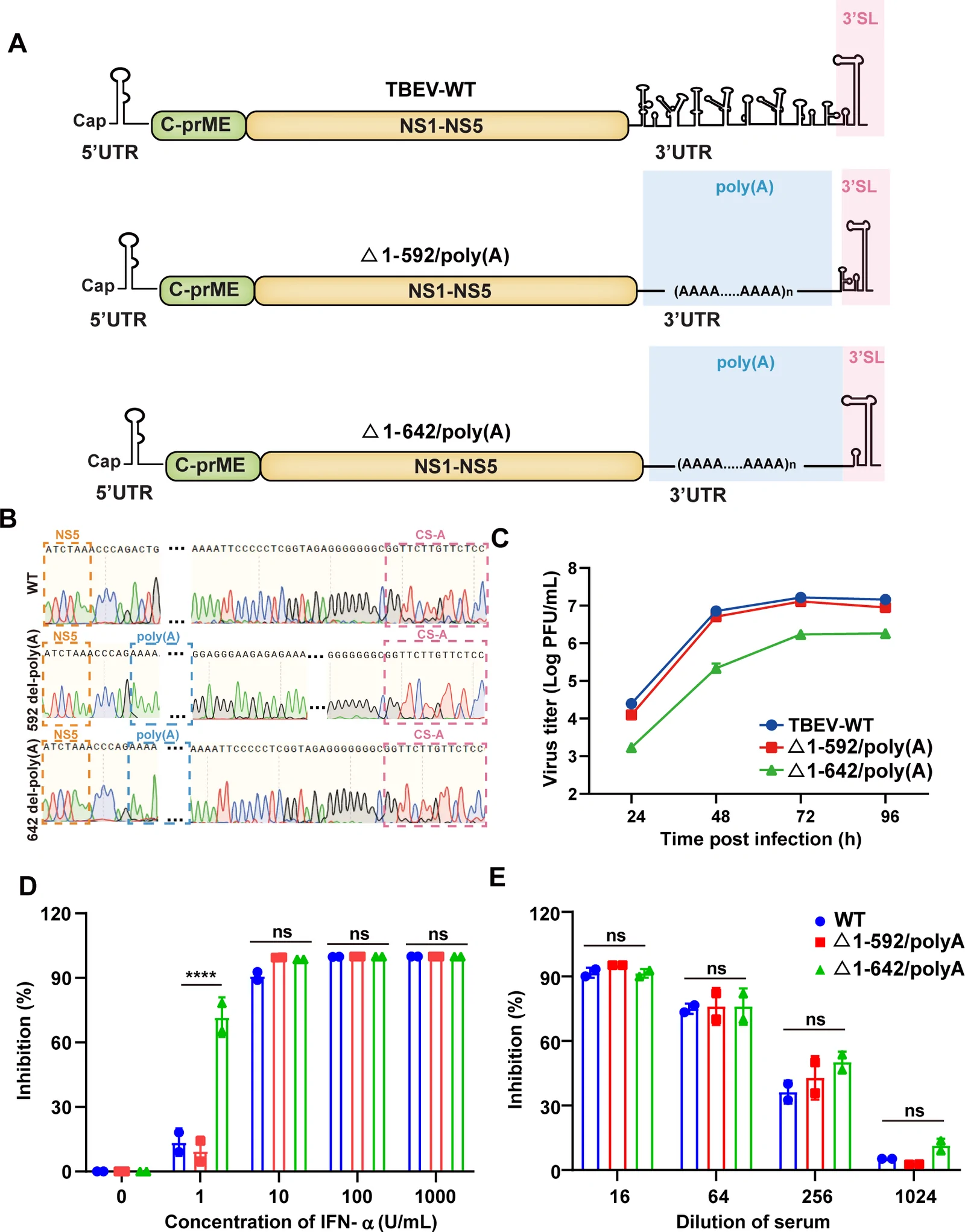
Engineering and virological characteristics of Δ1-592/poly(A) and Δ1-642/poly(A) mutant viruses. (**A**) Schematic representation of the TBEV-WT, Δ1-592/poly(A), or Δ1-642/poly(A) clones. WT 3′ UTR is represented by the second structure diagrams, and sequence deletion with poly(A) insertions is indicated by blue boxes. (**B**) Sequence chromatograms of WT TBEV, Δ1-592/poly(A), and Δ1-642/poly(A) in the 3′ UTR region. (**C**) Growth kinetics of WT TBEV, Δ1-592/poly(A), or Δ1-642/poly(A) in BHK-21 cells. BHK-21 cells were infected with WT TBEV, Δ1-592/poly(A), or Δ1-642/poly(A) at an MOI of 0.1. Supernatant was collected at 24, 48, 72, and 96 h post-infection (hpi), and the viral titers were determined by focus-forming assay. (**D**) Sensitivity of WT TBEV, Δ1-592/poly(A), and Δ1-642/poly(A) to interferon α (IFN-α) treatment in BHK-21 cells. BHK-21 cells were infected with either WT TBEV, Δ1-592/poly(A), or Δ1-642/poly(A) at an MOI of 0.1 and treated with different concentrations of IFN-α. The supernatants were harvested at 48 hpi, and the viral titers were measured by focus-forming assay. (**E**) Neutralizing activity of anti-serum against TBEV-WT, Δ1-592/poly(A), and Δ1-642/poly(A). Viruses were incubated with anti-serum from mice infected with WT viruses, and focus reduction neutralization assay was performed. All the experiments were done twice in duplicates. The data shown were the average values of the duplicates from a representative experiment with standard deviation (error bars). ****P < 0.0001; n.s., no significant differences.

Following the same method as previously reported (17), the clone pool of full-length Δ1-592/poly(A) or Δ1-642/poly(A) with different lengths of internal poly(A) sequences was constructed, and *in vitro* transcribed genome RNA pools were transfected into BHK-21 cells. The recombinant viruses were passaged in BHK-21 cells, and increased amounts of IFA-positive cells were observed (Fig. S2A and C), indicating the successful recovery of TBEV Δ1-592/poly(A) and Δ1-642/poly(A) mutant viruses (Fig. 2A). Complete genome sequencing of passaged viruses confirmed that approximately 300 nt of poly(A) tract was successfully inserted into the genome between the NS5 coding region and 3′ UTR sequences as expected (Fig. 2B). *In vitro* growth kinetics analysis in BHK-21 cells showed that both Δ1-592/poly(A) and Δ1-642/poly(A) viruses exhibited efficient propagation, and Δ1-592/poly(A) replicated similarly to the WT virus, while the multiplication of Δ1-642/poly(A) was about 10-fold slower than that of WT, with a peak titer of 10^6^ FFU/mL at 72 h post-infection (Fig. 2C). Moreover, to test the genetic stability of Δ1-592/poly(A) and Δ1-642/poly(A), three independent viral stocks (a, b, and c) were subjected to 5 or 10 rounds of blind passaging in BHK-21 cells. The RT-PCR amplicons of 3′ UTR from passaged mutants were similar to that of P1 products (Fig. S2B and D), and the sequencing results confirmed the poly(A) tracts and sequence deletion in 3′ UTR were still maintained (Fig. S2E). Moreover, to further verify whether there are any other mutations, complete genome sequencing of P10 Δ1-642/poly(A) was performed, and the results showed that multiple mutations emerged, predominantly located within non-structural protein regions (Fig. S2F). We speculate that these substitutions appear to arise randomly, as no shared mutations were identified across all three independently passaged viral lineages, which may not alter the antigenicity of the virus.

Our previous study has shown that WNV-poly(A) was more susceptible to interferon (IFN)-α than that of WT (17). To further verify this result, we conducted a similar IFN experiment, and the results demonstrated that TBEV Δ1-642/poly(A) mutant exhibited greater sensitivity to IFN treatment compared with WT TBEV. As shown in Fig. 2D, the viral titers of Δ1-642/poly(A) were dramatically reduced relative to WT or Δ1-592/poly(A) at interferon-α concentrations of 1 U/mL (~70%). Moreover, viral titers of the Δ1-642/poly(A) mutant failed to efficiently elevate and remained markedly lower than WT at all post-infection time points in IFN-I competent A549 and Huh7 cells (Fig. S3), further verifying the vulnerability of Δ1-642/poly(A) mutant to type I IFN. Furthermore, Δ1-592/poly(A) and Δ1-642/poly(A) shared similar antigenic profiles with the WT virus. Specifically, sera from mice infected with WT TBEV inhibited the infection of both mutant viruses and WT TBEV to a comparable extent (Fig. 2E). Collectively, these data demonstrate that the Δ1-592/poly(A) and Δ1-642/poly(A) mutants possess antigenic activity equivalent to that of WT TBEV and Δ1-642/poly(A) showed enhanced sensitivity to interferon—characteristics that support the potential as live-attenuated vaccine candidates.

### TBEV Δ1-642/poly(A) is highly attenuated in outbred ICR mice

**Fig 3:**
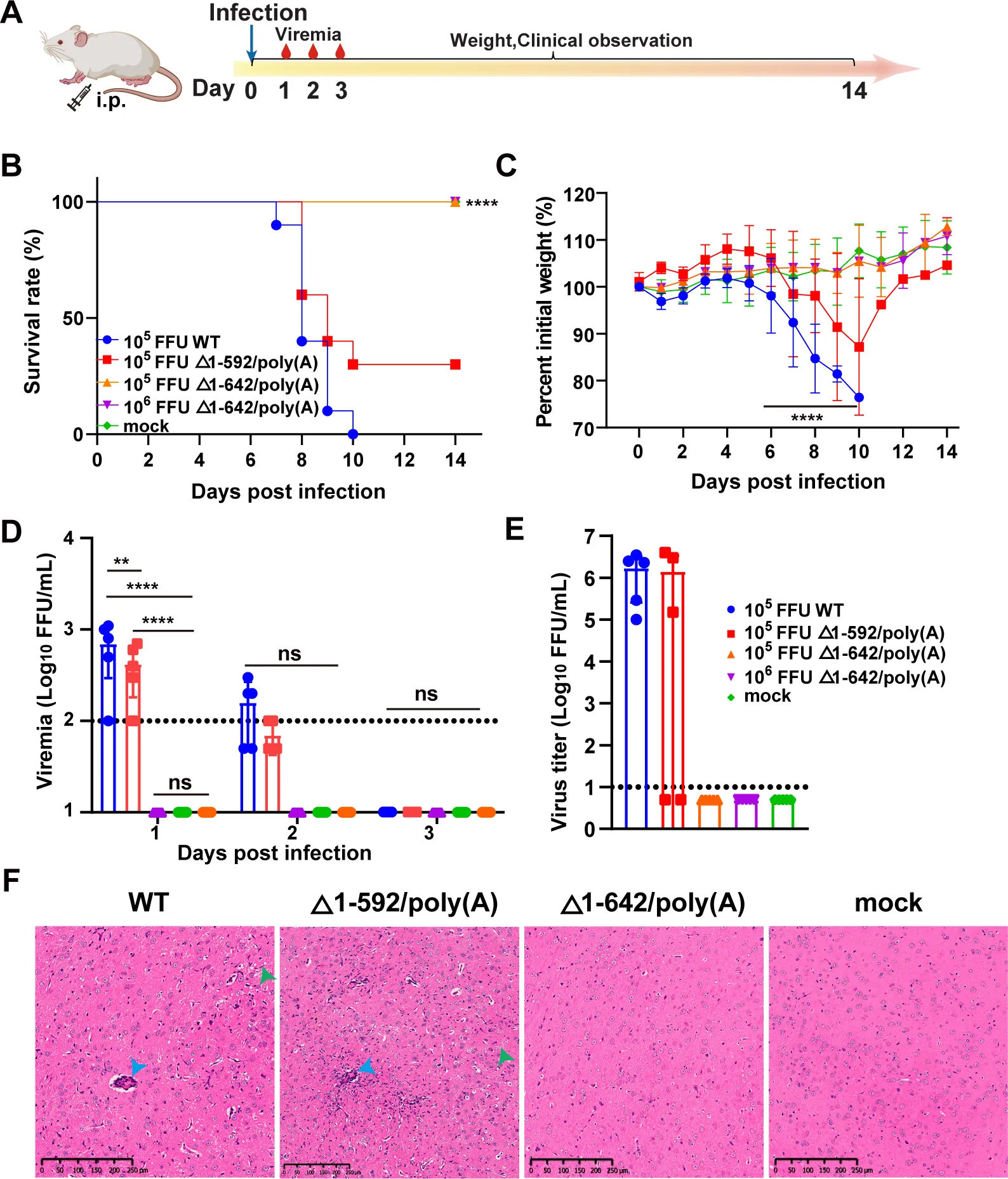
Virulence of Δ1-592/poly(A) and Δ1-642/poly(A) in outbred ICR mice. (**A**) Flowchart of virulence experiments. Four-week-old outbred ICR mice (*n* = 5) were intraperitoneally (i.p.) inoculated with 10^5^ FFU of WT TBEV, Δ1-592/poly(A), or 10^5^ or 10^6^ FFU of Δ1-642/poly(A). (**B**) Survival analysis of mice injected with WT, Δ1-592/poly(A), or Δ1-642/poly(A) viruses for 14 days post-infection. (**C**) Body weight changes of ICR mice infected with TBEV WT or mutant viruses were monitored for 14 days. (**D**) Viremia was measured by focus-forming assay on days 1, 2, and 3 after infection. The dotted line indicates the detection limit (100 FFU/mL). (**E**) Virus loads in the brains of mice infected with WT or mutant viruses at 7 dpi. The values represent mean ± SD in each group; each symbol represents a mouse. ****P < 0.0001, ns, no significant difference. (**F**) Pathological changes in the brain tissues of mice following WT TBEV or mutant viruses’ infection. Severe vacuolization (green arrows) and inflammation (blue arrow) in brain small vessels were indicated. Scale bars were marked.

To evaluate the virulence of Δ1-592/poly(A) and Δ1-642/poly(A), groups of 4-week-old outbred ICR mice (*n* = 5) were intraperitoneally (i.p.) inoculated with WT (10^5^ FFU/mouse), Δ1-592/poly(A) (10^5^ FFU/mouse), or Δ1-642/poly(A) (10^5^ or 10^6^ FFU/mouse) viruses. All the infected mice were observed daily for clinical signs of illness and survival rate for 14 days, and viremia was detected within 3 days post-injection (Fig. 3A). Mice infected with WT virus started to develop severe symptoms, including ruffled fur, limb paralysis, and weight loss at 6 days post-injection (Fig. 3C), and all mice were sacrificed after 10 days post-injection (Fig. 3B). Moreover, the Δ1-592/poly(A) virus-infected mice developed symptoms after 7 dpi, with an average mortality rate of 60% throughout the observation period. In contrast, the mice infected with Δ1-642/poly(A) displayed no weight reduction and 100% survival (Fig. 3B).

In addition, we quantified serum viremia from days 1 to 3 post-inoculation. For WT virus-infected mice, viremia peaked at day 1 and dropped sharply on days 2 and 3. The Δ1-592/poly(A) group displayed marginally reduced viremia at day 1 compared with the WT group. In contrast, viremia remained nearly undetectable across the entire observation period in mice inoculated with Δ1-642/poly(A) (Fig. 3D). Given that TBEV predominantly induces encephalitis and exhibits strong tropism for the brain, we quantified brain viral loads and characterized pathological changes using H&E staining at 7 days post-infection (dpi). As shown in Fig. 3E, viral loads in the brains of mice infected with the WT virus and moribund mice infected with Δ1-592/poly(A) were comparable, whereas no infectious viruses were detected in the Δ1-642/poly(A) group. Histological analysis revealed that the brain tissue from WT- or Δ1-592/poly(A)-infected moribund mice exhibited severe vacuolization (green arrows) and inflammation in brain small vessels (blue arrow), as well as neuronal necrosis, degeneration, and disruption of normal cytoarchitecture, as depicted in Fig. 3F. While consistent with the observed clinical phenotypes, no obvious brain lesions were observed in Δ1-642/poly(A)-infected mice in comparison with mock-infected mice. Moreover, the viral loads of other tissues in moribund WT or Δ1-642/poly(A)-infected mice at 7 dpi were measured, and no live viruses were detected in all Δ1-642/poly(A)-infected mice. Meanwhile, in WT-infected mice, infectious viruses could be detected in all or most tissue collected (Fig. S4). These results implied that Δ1-642/poly(A) exhibited greater attenuation compared to WT and Δ1-592/poly(A) in ICR mice, suggesting its potential for development as a LAV.

### A single dose of Δ1-642/poly(A) induces robust humoral and cellular immune responses and protects ICR mice from lethal WT TBEV challenge

**Fig 4:**
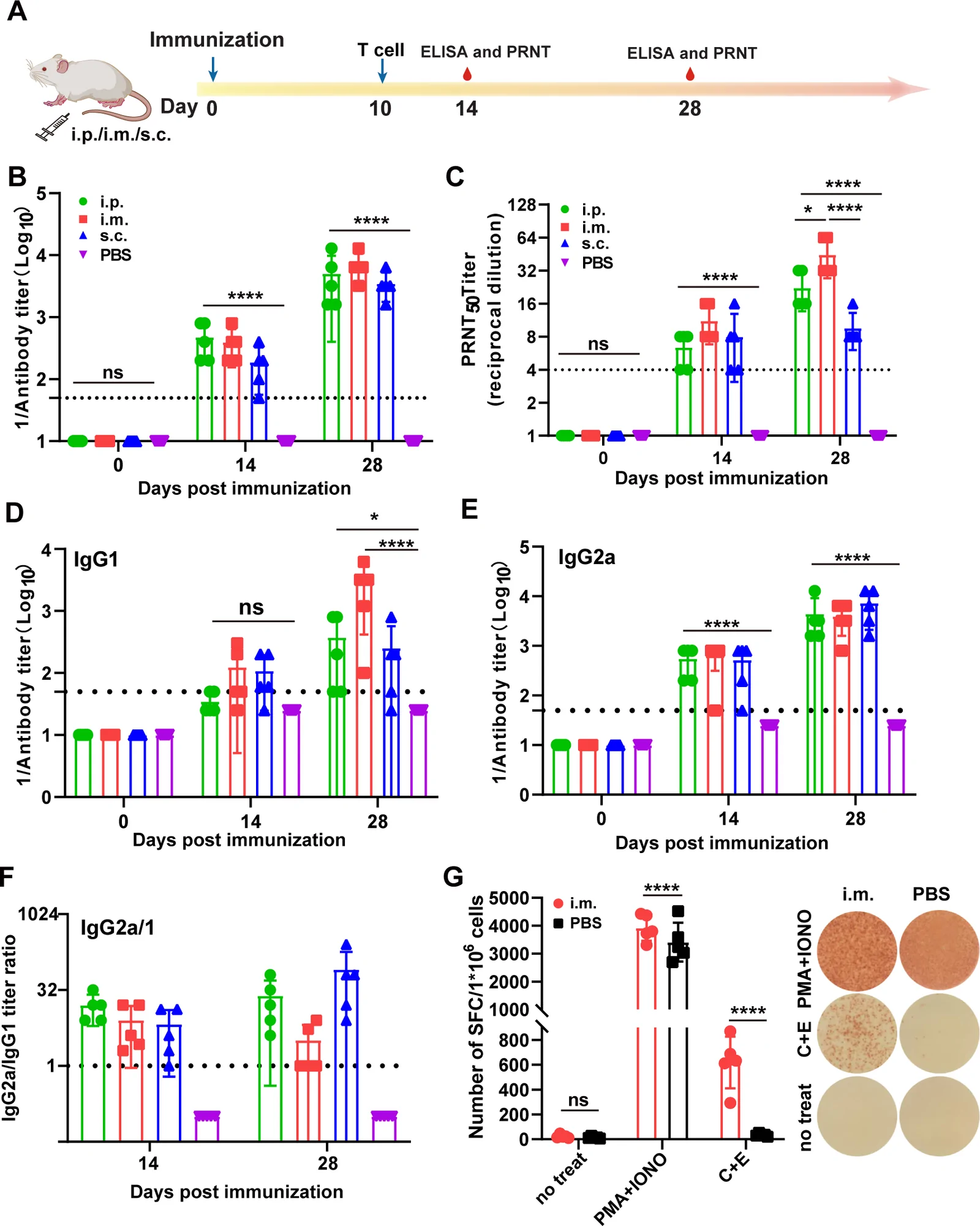
Immunogenicity of Δ1-642/poly(A) in ICR mice. (**A**) Experimental scheme of immunization in mice. Groups of 4-week-old outbred ICR (*n* = 5) mice were immunized with 10^6^ FFU of Δ1-642/poly(A) or PBS (negative control) through intraperitoneal (i.p.), intramuscular (i.m.), or subcutaneous (s.c.) route. At 10 days post-immunization, splenocytes were collected for T-cell immune responses detection and the serum of 14 or 28 days post-immunization were collected for IgG and neutralizing antibody analysis. (**B and C**) Total anti-TBEV IgG antibody (**B**) and neutralizing antibody titers (**C**) in mouse serum at 14 or 28 days post-immunization. (**D through F**) IgG subtype analysis by ELISA. The serum samples from each group were subjected to ELISA assay to measure the antibody levels of IgG1(**D**) and IgG2a (**E**), and IgG2a/IgG1 ratios (**F**) were calculated and presented. (**G**) T-cell responses in vaccinated mice were measured through ELISpot assay. Two groups of ICR mice (*n* = 5/group) were i.m. inoculated with 10^6^ FFU of Δ1-642/poly(A) or an equal volume of PBS. ELISpot assay specific for IFN-γ in splenocytes was performed at 10 days post-immunization. Representative images were shown, and the numbers of spot-forming cells (SFCs) were counted (the values represent mean ± SD in each group; each symbol represents a mouse). *P < 0.05, ****P < 0.0001, ns, no significant difference.

To assess the potential of Δ1-642/poly(A) as a LAV, 4-week-old outbred ICR mice (*n* = 5) were inoculated once with 10^6^ FFU of Δ1-642/poly(A) via i.p., intramuscular (i.m.), or subcutaneous (s.c.) routes (Fig. 4A). Total IgG antibody and neutralizing antibody titers against TBEV were measured with the sera from immunized mice on days 14 and 28 using enzyme-linked immunosorbent assay (ELISA) and focus reduction neutralization test, respectively. As shown in Fig. 4B, robust IgG antibody responses were induced in all Δ1-642/poly(A)-immunized groups by day 28, with average titers reaching 1:5,120, 1:7,000, and 1:3,520 in the i.p., i.m., and s.c. groups, respectively. Consistently, neutralizing antibody titers at day 28 were 1:22.4, 1:44.8, and 1:9.6 for the different immunization routes, with the i.m. route eliciting significantly higher neutralizing antibody levels than either the i.p. or s.c. routes (Fig. 4C). Furthermore, both IgG1 and IgG2a were significantly elicited by 14 and 28 days post-immunization, but IgG2a titers exceeded those of IgG1, resulting in an IgG2a/IgG1 ratio greater than 1 (Fig. 4D through F), indicating a Th1-biased immune response.

To further assess T-cell responses in immunized mice, IFN-γ ELISpot assay was performed to detect the number of TBEV-specific IFN-γ-secreting cells on day 10 after immunization with Δ1-642/poly(A) (Fig. 4G). The splenocytes were isolated from the spleen of immunized mice and stimulated with the peptide pool corresponding to TBEV capsid and envelope proteins (C + E). Nonspecific stimulation of PMA and ionomycin (PMA + IONO) was used as a positive control, and medium alone served as a negative control. Much higher number of IFN-γ-secreting cells were observed in the Δ1-642/poly(A)-immunized mice compared to mock immunized mice (619 vs 35 per 1 × 10^6^ splenocytes; *P* < 0.0001) in response to C + E peptide pool stimulation (Fig. 4G). Taken together, our data suggested that Δ1-642/poly(A) could induce strong IFN-γ T-cell responses in mice after a single-dose inoculation.

**Fig 5:**
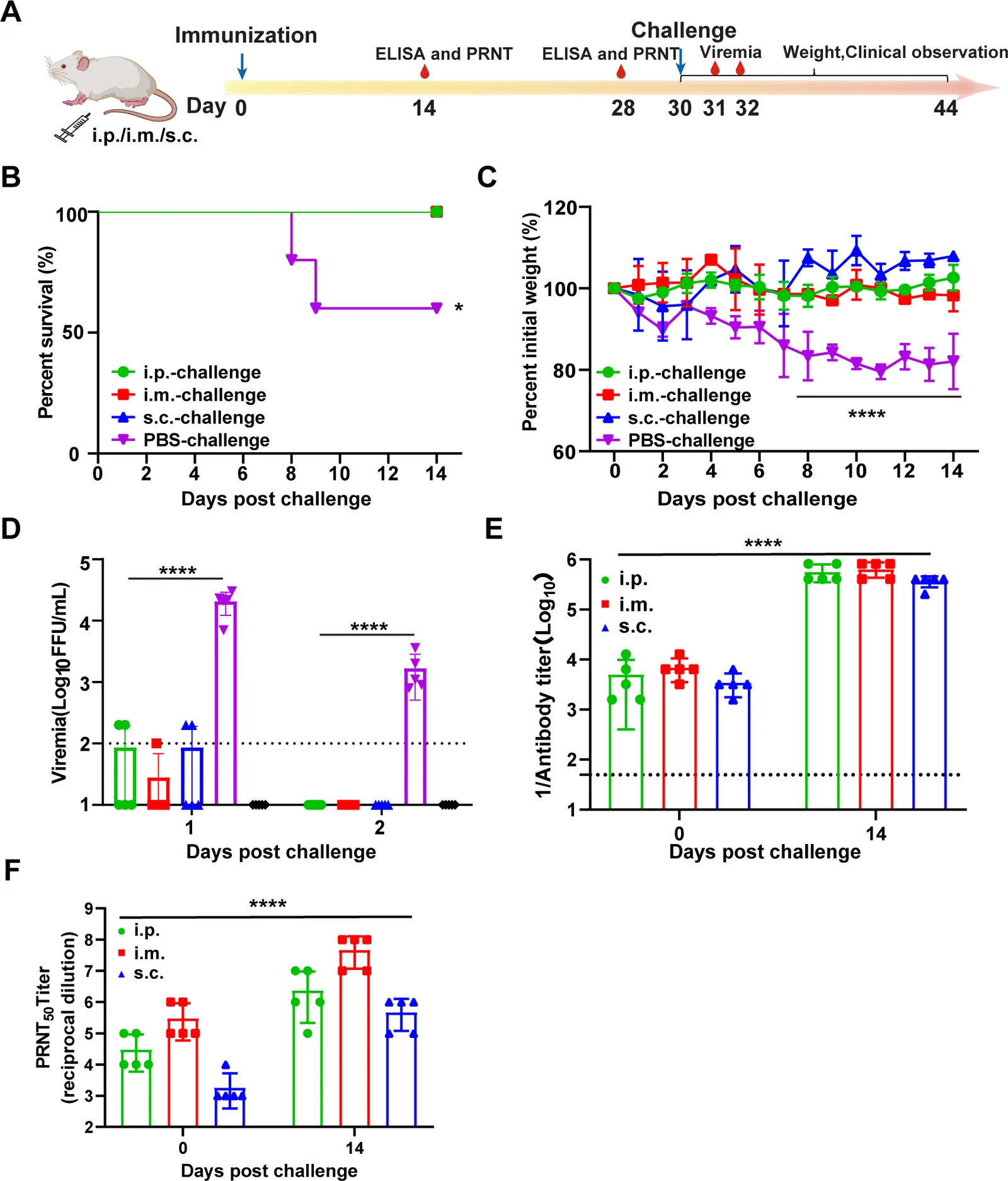
Immune protection of Δ1-642/poly(A) against lethal WT TBEV challenge in ICR mice. (**A**) Experimental scheme of immunization and WT virus challenge in mice. On day 30 post-immunization, mice (*n* = 5) were challenged i.p. with 10^7^ FFU of WT TBEV. Survival (**B**) and body weight changes (**C**) were monitored daily after challenge. (**D**) Viremia was quantified by focus-forming assay from day 1 or 2 post-challenge. The values represent mean ± SD in each group, and the horizontal dotted line represents the limit of detection. (**E and F**) The serum samples from the Δ1-642/poly(A) immunized and the challenged mice were subjected to ELISA or focus reduction neutralization assay to measure the antibody levels of IgG (**E**) and neutralizing antibodies (**F**). The values represent mean ± SD in each group; each symbol represents a mouse. *P < 0.05, ****P < 0.0001.

At 30 days post-immunization, mice from each group (*n* = 5) were challenged with 10⁷ FFU of WT virus via the i.p. route to evaluate protective immunity (Fig. 5A). All mice immunized with Δ1-642/poly(A) were fully protected against the challenge, surviving without any signs of illness, body weight loss, or viremia (Fig. 5B through D). In contrast, all mock-immunized mice (PBS-treated) developed severe illness or succumbed to death, accompanied by weight loss and high viremia levels. Moreover, comparing with pre-challenge, the titers of TBEV-specific IgG antibodies and neutralizing antibodies in Δ1-642/poly(A) vaccinated mice increased about 100-fold (Fig. 5E) and fourfold (Fig. 5F), respectively, indicating that the TBEV challenge potentiates the antibody responses of the immunized mice.

Moreover, a group of mice (*n* = 5) from i.m. immunization was challenged with 1 FFU of WT virus via the intracranial (i.c.) route to evaluate the protection against neurovirulence. The results showed that the Δ1-642/poly(A)-immunized group exhibited a 60% survival rate, whereas all PBS control mice succumbed to infection within 10 days post-challenge (Fig. S5), demonstrating a single Δ1-642/poly(A) immunization could partially protect ICR mice against direct neurovirulence. Collectively, our results demonstrated that a single dose of Δ1-642/poly(A) immunization can achieve robust immune responses and protect ICR mice against the lethal WT virus challenge.

### A single dose of Δ1-642/poly(A) immunization induced long-term immune responses and protected ICR mice from lethal WT TBEV challenge

**Fig 6:**
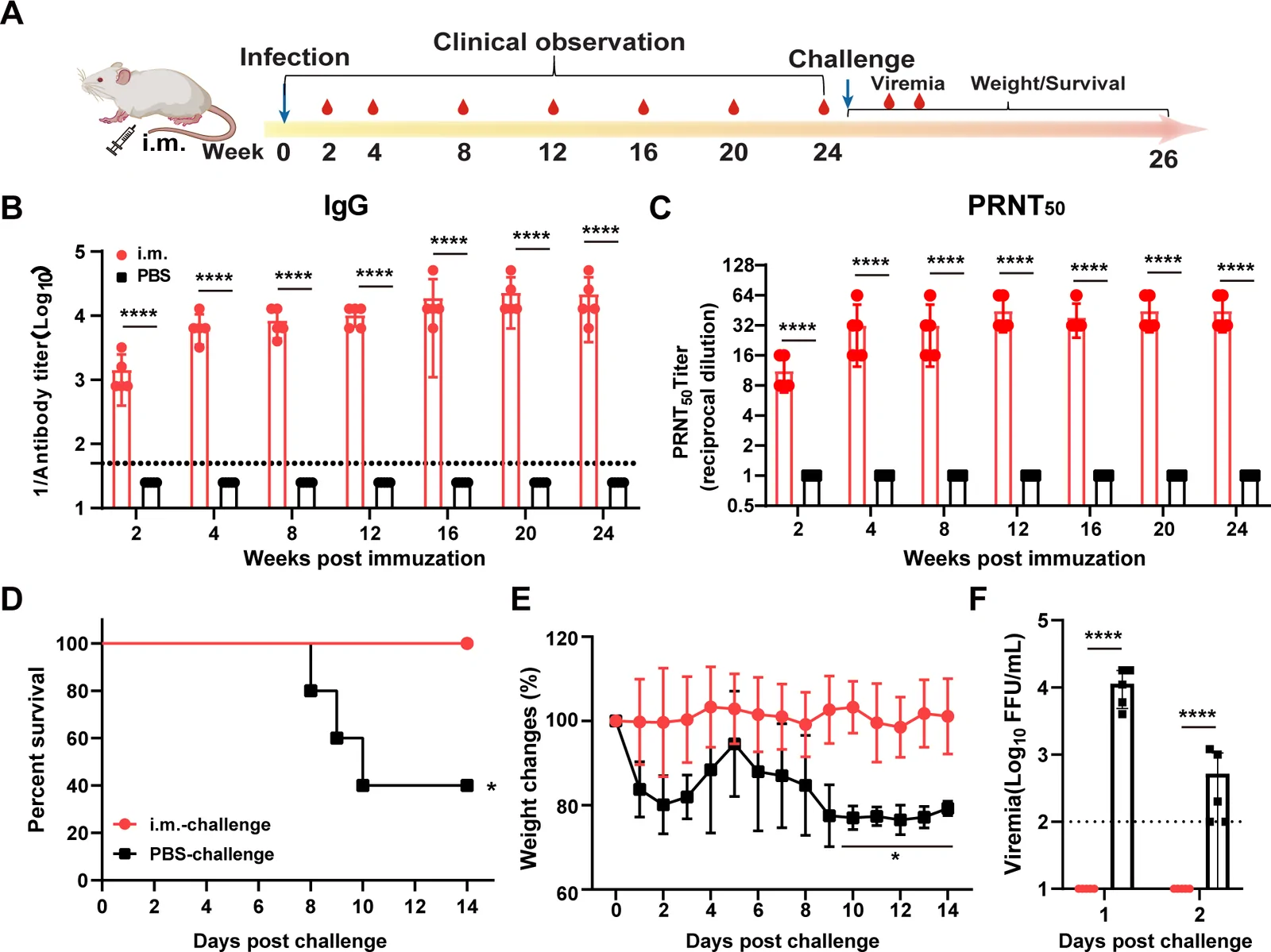
Long-term antibody responses induced by a single-dose (10^6^ FFU) of Δ1-642/poly(A) protect mice from lethal WT virus challenge. (**A**) Experimental scheme of long-term immunization and WT virus challenge in ICR mice. Groups of 4-week-old outbred ICR mice (*n* = 5) were given a single-dose i.m. injection of 10^6^ FFU Δ1-642/poly(A) or PBS. The serum samples of mice from each group were collected at 2, 4, 8, 12, 16, 20, and 24 weeks after immunization, and then the mice were challenged with 6 × 10^6^ FFU of WT TBEV. The immunized serum was subjected to ELISA and focus reduction neutralization assays to quantify the levels of TBEV-specific antibodies (**B**) and neutralizing antibodies (**C**), respectively. The dashed lines represent the limit of detection. (**D–F**) After challenge, the survival (**D**) and body weight changes (**E**) of mice were monitored daily for another 2 weeks. (**F**) Viremia was quantified by focus-forming assay from day 1 or 2 post-challenge. The values represent mean ± SD in each group, and the horizontal dotted line represents the limit of detection. *P < 0.05, ****P < 0.0001.

To further examine the durability of protective immunity, another group of ICR mice (*n* = 5) inoculated through the i.m. route with a single dose of 10^6^ FFU Δ1-642/poly(A) was not challenged and maintained for 24 weeks, and the titers of TBEV-specific IgG antibodies and neutralizing antibodies were continuously analyzed at 4-week intervals post-immunization (Fig. 6A). Then, the immunized mice were challenged with 6 × 10^6^ FFU of WT virus through i.p. route. It showed that both titers of TBEV-specific IgG and neutralizing antibodies were maintained at a high level throughout the whole experimental period in Δ1-642/poly(A)-vaccinated ICR mice (Fig. 6B and C). Furthermore, all the immunized mice showed no adverse symptoms throughout the experimental period, further confirming the safety profile of the vaccine. In addition, after the WT virus challenge, all PBS-treated mice developed severe illness or succumbed to death, accompanied by weight loss and high viremia levels, but all the mice immunized with Δ1-642/poly(A) survived without any signs of illness, body weight loss, or viremia (Fig. 6D through F). These results suggest that a single-dose immunization of Δ1-642/poly(A) can induce long-term robust immune responses and protect ICR mice against lethal TBEV infection.

## DISCUSSION

Vaccines are one of the most effective means for preventing and controlling infectious diseases, which play a crucial role in reducing disease transmission rates, alleviating disease severity, protecting susceptible populations, and reducing the pressure on medical resources. Currently, only inactivated vaccines against tick-borne encephalitis virus (TBEV) have been approved, but the vaccines normally require multiple immunizations. Meanwhile, the live-attenuated vaccine (LAV) mimics natural infection, and a single immunization could generate a robust and long-term immune response. Targeting the 3′ UTR without altering viral antigenicity offers an intriguing approach for the design of live-attenuated orthoflavivirus vaccines. Currently, a clinical trial is underway for the tetravalent vaccine candidate Butantan dengue vaccine, which features a 30-nucleotide deletion in the DB2 region of the 3ʹ UTR (21, 22). This development further motivates us to explore a universal vaccine strategy through 3ʹ UTR modification.

In our previous research, we developed a new WNV LAV candidate, WNV-poly(A), by inserting internal poly(A) stretches through extensive replacement of the variable segment within the 3′ UTR (17). Here, we demonstrated that the internal poly(A) tracts replacement strategy is equally viable for developing a LAV of TBEV. TBEV Δ1-642/poly(A) viruses exhibited significant attenuation, with no live viruses detected in the brain and other tissues, and a single immunization induced robust neutralizing antibody and T-cell immune responses and provided complete protection against the WT virus challenge in outbred ICR mice models (Fig. 3 to 5). The study describes a promising strategy for improving immunity against TBEV infection, which could serve as a potential vaccination approach for combating TBEV. Our research on TBEV Δ1-642/poly(A) and WNV-poly(A) confirmed the feasibility of employing the poly(A) insertion and replacement strategy as a new universal approach for the development of both mosquito-borne and tick-borne orthoflavivirus LAVs.

Consistent with previous studies on WNV, the whole variable region and most of the conserved region within TBEV 3′ UTR were lethal and can be fully substituted with poly(A) insertions, and the poly(A) tract insertion not only facilitates efficient viral replication but also attenuates the TBEV virulence. The findings of this study further validate our previous hypothesis regarding orthoflaviviruses: the length of the nucleotide sequence, rather than the specific primary sequence in the 5′ region of 3′ UTR, is the key to determining efficient viral replication (17). The 3′ UTR sequence length of TBEV (762 nt) was indeed longer than that of WNV (631 nt), which might be the reason why the poly(A) tract of TBEV Δ1-642/poly(A) (~300 nt) was longer than that of WNV-poly(A) (~130 nt). Moreover, the Δ1-592/poly(A) and Δ1-642/poly(A) mutant viruses exhibited significant differences in replication, sensitivity to IFN-α, and virulence (Fig. 2 and 3), suggesting that the AU-SL structure in the conserved region may serve as a key element regulating viral replication and virulence.

Notably, our observations differ from prior investigations that characterized the poly(A) tract as a virulence determinant for TBEV in mice. Specifically, the Torö−2003 strain harboring a 38A tract within the variable region of 3′ UTR exhibited enhanced virulence relative to its counterpart containing a 6A tract (18). We hypothesize that this discrepancy in findings may arise from variations in the preservation of the 5′ complete region of 3′ UTR across studies (15, 19). It is conceivable that the internal insertion of 38A within the variable region of the Torö−2003 strain (18) alters the RNA secondary structure, thereby promoting interactions with host factors and ultimately increasing the viral virulence. However, our strategy involving poly(A) tract insertion and majority 5′ sequence deletion may weaken or alter the interactions with host factors. Further research is required to clarify the attenuation mechanism mediated by poly(A) substitution coupled with extensive sequence deletion.

In addition, previous studies on candidate TBEV vaccines have explored multiple strategies, including virus-like particles (VLPs) (23, 24), MVA vectors encoding prME (25), and Langat virus (LGTV) chimeras designated T/1674-mirV2, which were constructed via prME replacement and microRNA insertion (26). All these platforms elicit potent immune responses and confer full protection against the lethal WT TBEV challenge in mice. Apart from the T/1674-mirV2 constructs, all remaining vaccine candidates—including the licensed commercial vaccine Ticovac (FSME-IMMUN)—necessitate two or three booster doses. In contrast, the two LAV candidates, T/1674-mirV2 and our Δ1-642/poly(A) mutant examined herein, induce potent immune protection following only a single immunization. Notably, mice receiving a single dose of T/1674-mirV2 produced neutralizing antibody (NA) titers of approximately 1:1,000 (26), substantially exceeding those induced by Δ1-642/poly(A). This discrepancy likely stems from differences in the cell lines utilized for neutralization assays. Even though the NA titers measured in our Δ1-642/poly(A) immunization groups were relatively modest, they still surpassed the established minimal protective threshold of 1:10 (27) and sufficient to shield mice from the lethal TBEV infection.

In summary, our data revealed the potential of the new attenuation strategy vaccine Δ1-642/poly(A) to combat TBEV infection in outbred ICR mice. However, there are several limitations in this study that need to be solved in our future research. First, the mechanism underlying the attenuation of Δ1-642/poly(A) remains unclear. While increased sensitivity to IFN-α may facilitate viral clearance, the nature of interactions between the 3′ UTR sequence and host factors requires further investigation. Furthermore, constraints regarding the study’s scope and experimental setup precluded a direct head-to-head evaluation of efficacy relative to commercially approved inactivated TBEV vaccines, namely, FSME-IMMUN or Ticovac. Overall, this work is a proof-of-concept study for Δ1-642/poly(A) as a potential vaccine candidate against TBEV, and extensive studies including the optimization and scale-up of production of the vaccine, as well as pre-clinical studies on the safety and efficacy of the vaccine in other animal models, are required to facilitate clinical development of the vaccine.

## MATERIALS AND METHODS

### Cell lines, viruses, and antibodies

BHK-21, A549, and Huh7 cells were cultured in Dulbecco’s modified Eagle’s medium (DMEM) (Gibco, USA) with 10% fetal bovine serum (FBS) and 100 units penicillin/mL^−1^ and 100 µg streptomycin/mL^−1^ (PS) at 37°C in 5% CO_2_. TBEV WT (strain Neudoerfl, GenBank no. U27495) or mutant viruses were obtained by linearization of plasmids, *in vitro* transcription, and transfection into cells. The 4G2 antibody against the E protein of flavivirus was kindly provided by professor Shengbo Cao in Huazhong Agricultural University and cross-reactive with the TBEV E protein. HRP- or FITC-conjugated goat anti-mouse IgG was purchased from Protein Tech Group, China.

### Plasmid construction

All plasmids were constructed using standard molecular biology protocols and confirmed by DNA sequencing. The full-length infectious cDNA clone of TBEV WT was constructed by reverse transcription-amplification of viral gene sequences, which were then ligated into the pACYC177 vector, designated as pACYC‐TBEV-WT. Using pACYC-TBEV-WT as the backbone, a series of 3′ UTR deletion mutants were introduced by fusion PCR, and the sequences of the primers used in the construction of the mutants were listed in Table S1. The K*pn*I and N*ot*I restriction sites were used to construct the infectious clones of deletion mutants. The Δ1-592/poly(A) or Δ1-642/poly(A) cDNA libraries were constructed as described previously (17). Briefly, the fragment A covering the 3′ part of NS5 was inserted into the pMD18T vector and subjected to *in vitro* transcription, poly(A) tail addition, and RT-PCR. At the same time, fragment B containing poly(A) and 3′ SL sequences was amplified. The fusion PCR products of fragments A and B and the fragment L covering the 5′ UTR to NS5 were *in vitro* ligated at 4°C overnight, and ligation DNA was purified by phenolic chloroform extraction for *in vitro* transcription. The primers used for fragment amplification are shown in Table S1.

### *In vitro* transcription, RNA transfection, virus rescue, and sequencing

TBEV WT and deletion mutant (Δ1-509, Δ1-592 and Δ1-642) clones were linearized with NotI. The linearized cDNA was *in vitro* transcribed using the mMESSENGER mMACHINE T7 Kit (Ambion, Austin, TX, USA). All procedures were performed according to the manufacturer’s protocols. RNA was dissolved in RNase-free water and stored at −80°C. 1 µg of RNA was transfected into BHK-21 cells with DMRIE-C reagent (Invitrogen, USA) following the manufacturer’s protocols. The expression of E protein was detected by IFA. Three parallel strains of the Δ1-592/poly(A) or Δ1-642/poly(A) mutant supernatants were subjected to serial passage in BHK-21 cells until P5 or P10 passages, respectively. The passaged viruses were subjected to RT-PCR and Sanger sequencing to test the 3′ UTR. In addition, full-length genome sequencing of P10 Δ1-642/poly(A) was performed through segmented RT-PCR and Sanger sequencing. The primers used are listed in Table S1.

### Indirect immunofluorescence assay (IFA)

The transfected or infected cells were fixed with ice-cold 5% acetone in methanol for 10 min at room temperature, washed three times with PBS, and then incubated with the 4G2 antibody (1:500 dilution in PBS) at 37°C for 1 h. After washing three times with PBS, the cells were incubated with secondary antibodies conjugated to FITC (1:125 dilution in PBS) for 40 min in the dark. After washing three times with PBS, the cells were mounted on a glass slide with 95% glycerol, and cell images were captured under a fluorescence microscope (Zeiss).

### Focus-forming assay and viral growth curves

The virus titer of TBEV WT, Δ1-592/poly(A), or Δ1-642/poly(A) was determined by focus-forming assay. According to the conventional plaque assay, BHK-21 cells were infected with the viruses of serially diluted 10-fold, and incubated with DMEM-2% FBS containing 1% methylcellulose for 1 day in a 24-well plate. Then, the cells were fixed with ice-cold 5% acetone in methanol, and then IFA was performed as described above. Positive cell clusters were counted under a fluorescence microscope, and viral titers were calculated as focus-forming units per mL (FFU/mL).

For viral growth curve analysis, BHK-21 cells were infected with WT or Δ1-592/poly(A) or Δ1-642/poly(A) viruses at a multiplicity of infection (MOI) of 0.1, and the supernatants were harvested at the indicated time points and stored at −80°C. The viral titers at different time points were determined by the focus-forming assay. The growth curves were plotted using GraphPad Prism 8.0 software.

### IFN‐α treatment

BHK-21 cells seeded in 12‐well plates (1.2 × 10^5^ cells per well) were infected with TBEV-WT or Δ1-592/poly(A) or Δ1-642/poly(A) virus at a MOI of 0.1 in the presence of different concentrations of IFN‐α. At 48 h post‐treatment, the supernatants containing different concentrations of IFN‐α were harvested, and viral titers were measured by focus-forming assay.

### Mouse studies

To test the pathogenesis of poly(A) viruses, 4-week-old female ICR mice (*n* = 5 per group) were inoculated intraperitoneally (i.p.) with 10^5^ or 10^6^ FFU of poly(A) viruses, 10^5^ FFU of WT TBEV, or PBS (negative control). The infected mice were monitored daily for survival, viremia, and body weight changes. Viremia was detected after 1, 2, and 3 days post-infection by focus-forming assay. At 7 days post-infection (dpi), brains were harvested to test viral load and made into paraffin sections followed by H&E staining, and different tissues including heart, liver, spleen, lung, and kidney were also harvested to test viral loads.

For immunization and challenge experiments, groups of 4-week-old ICR mice (*n* = 5 per group) were immunized with 10^6^ FFU of Δ1-642/poly(A), or PBS (negative control) through intraperitoneally (i.p.), intramuscular (i.m.), or subcutaneous (s.c.) routes. At 14 or 28 days post-immunization, the titers of total anti-TBEV IgG antibodies and neutralizing antibodies in mouse serum were examined by enzyme-linked immunosorbent assay (ELISA) and focus reduction neutralization test, respectively. At 30 days post-immunization, mice were challenged i.p. or i.c. with 1 × 10^7^ or 1 FFU of WT TBEV, respectively. Survival and body weight changes were monitored daily until 14 days later. Viremia was quantified by focus-forming assay on day 1 or 2 post-challenge.

For long-term immunization detection, two groups of i.m. or PBS immunized mice (*n* = 5 per group) were maintained for 6 months (24 weeks). The titers of total anti-TBEV IgG antibodies and neutralizing antibodies in mouse serum were examined at 4-week intervals. Then, the immunized mice were challenged i.p. with 6 × 10^6^ FFU of WT TBEV. Survival and body weight changes were monitored daily until 14 days later. Viremia was quantified by focus-forming assay on day 1 or 2 post-challenge.

### Enzyme-linked immunosorbent assay (ELISA)

The TBEV WT antigen in 0.5 M Na_2_CO_3_-NaHCO_3_ buffer was coated on 96-well plates overnight at 4℃. The plates were then blocked with 5% skim milk at 37℃ for 2 h. Sera from the immunized mice were fourfold diluted with 2% skim milk and added to plates for 2 h incubation. After washing steps, HRP-conjugated goat anti-mice IgG, IgG1, or IgG2a antibody (1:5,000) was added for 1 h at 37℃. The plates were then incubated with a mixed substrate (two-component 3,3′,5,5′-tetramethylbenzidine (TMB) color development kit [Proteintech]). The optical density at 450 nm was detected and analyzed by a multimode microplate reader (Varioskan Flash, Thermo, USA) after the addition of 1 M H_2_SO_4_ to terminate the coloration reaction. The IgG, IgG1, or IgG2a antibody titers were defined as the highest dilution of sera that yielded an absorbance >2 fold over background values.

### Focus reduction neutralization test

Neutralizing activity was determined by the focus reduction neutralization test. Briefly, the serum was inactivated for 30 min at 56°C. Serial twofold dilutions of inactivated serum were mixed with equal volumes of WT viral solution to yield a mixture containing ~50 FFU of virus per mL. After incubation at 37°C for 1 h, 100 μL volumes of the virus-serum mixtures were added to 24-well plates containing confluent monolayers of BHK-21 cells. Viral titers were then determined using the focus-forming assay described above. The endpoint neutralization titer was calculated as the highest dilution of serum yielding a 50% reduction in virus control focus numbers.

### Enzyme-linked immunospot (ELISpot) assay

IFN-γ ELISpot assay was performed using the commercial mouse IFN-γ ELISpot kit (2210006, Dakewe, China) following the manufacturer’s recommendations. Briefly, freshly isolated splenocytes from Δ1-642/poly(A)-immunized mice were plated at 1 × 10^6^ cells per well in the presence of TBEV C+E peptide pool (3672-1, Mab Tech, Sweden) at a final concentration of 1 µg/mL, or medium alone (negative control), or 4 µg/mL of PMA and ionomycin (positive control). After incubation at 37°C, 5% CO_2_ for 20 h, the plates were washed and incubated with biotinylated anti-IFN-γ antibody for 1 h at 37°C, followed by incubation with horseradish peroxidase (HRP)-conjugated streptavidin for 1 h at 37°C. The IFN-γ spots were developed using AEC substrate following the manufacturer’s instructions. After drying, the spots were scanned and counted using ELISPOT image analysis (Bio-Sys, Karben, Germany). The average of duplicate counts of IFN-γ spot-forming cells per well was calculated by subtracting background spots from negative controls.

### Statistical analysis

For viral burden analysis, an unpaired two-tailed *t* test was used to determine statistically significant differences. For three or more groups, variation between groups was measured by two-way analysis of variance with a multiple comparison test. For survival analysis, Kaplan–Meier survival curves were analyzed by a log-rank test. Statistical analyses were performed using standard GraphPad Prism software, version 8.0.

## Data Availability

All data supporting the findings of this study are available within the article or from the corresponding author upon reasonable request.

## SUPPLEMENTAL MATERIAL

Supplemental material jvi.00781-26-s0001.docx Fig. S1 to S5; Table S1.

ASM does not own the copyrights to Supplemental Material that may be linked to, or accessed through, an article. The authors have granted ASM a non-exclusive, world-wide license to publish the Supplemental Material files. Please contact the corresponding author directly for reuse.
