# Supplementary material for: Internal poly(A) replacement strategy within 3′ UTR for live-attenuated tick-borne encephalitis virus
Source: J Virol. 2026 Sep 16;100(10):e00781-26. doi: 10.1128/jvi.00781-26 (PMC13712986; doi:10.1128/jvi.00781-26)
Supplement: Supplementary file 1 [file jvi.00781-26-s0001.docx]

**Supplementary Files for**

**Internal poly(A) replacement strategy within 3ʹUTR for live-attenuated tick-borne encephalitis virus**

Qiu-Yan Zhang^1¶^, Zhe-Rui Zhang^1^^¶^, Cheng-Lin Deng^1¶^, Jing Wang, Hong-Qing Zhang, Han-Qing Ye, Ya-Nan Zhang^1,2*^, Bo Zhang^1*^

^1^State Key Laboratory of Virology and Biosafety, Wuhan Institute of Virology, Chinese Academy of Sciences, Wuhan, 430071, China.

^2^Hubei Jiangxia Laboratory, Wuhan 430200, China.

* Corresponding author

Email: [zhangbo@wh.iov.cn](mailto:zhangbo@wh.iov.cn); [ynzhang@wh.iov.cn](mailto:ynzhang@wh.iov.cn)

**This PDF file includes:**

Figure. S1-S5

Table S1

**Figure S1**

**Fig S1. Diagram of *orthoflavivirus* 3' UTR secondary structure.** WNV, DENV2 and ZIKV, belonging to mosquito-borne *orthoflavivirus* (MBFV), contain similar stem loop (SL) and dumbbell (DB) structure in 3' UTR. In TBEV Neudoerfl strain, there are Y-SL, AU-SL, and GC-SL structures within 3' UTR. sHP and 3ʹSL region (highlighted with pink box) is the most conserved region in all flaviviruses because of its crucial role in viral genome cyclization.

**Figure S2**

**Fig S2. Production and passaging of Δ1-592/poly(A) and** Δ**1-642/poly(A) mutant viruses in BHK-21 cells.** Δ1-592/poly(A) and Δ1-642/poly(A) mutant RNAs were transfected into BHK-21 cells and observed supernatants were passaged for three or five times, respectively, until the infectious viruses were produced. Then viruses were continuously passaged to P5 or P10. (A and C) Detection of E protein expression by IFA in BHK-21 cells infected with initial passaged viruses. (B and D) RT-PCR detection of 3' UTR of passaged viruses and the amplicons from P5 of Δ1-592/poly(A) and P10 of Δ1-642/poly(A) was similar to that of P1 products. (E) Sequence chromatograms of 3′ UTR of passaged P5 Δ1-592/poly(A) and P10 Δ1-642/poly(A). The chromatograms of WT TBEV were shown as reference. (F) The Sanger sequencing results of full length of genome of P10 Δ1-642/poly(A) mutant viruses. Genes without any mutations are not listed.

**Figure S3**

**Fig S3. The growth curves of TBEV WT and Δ1-642/poly(A) mutant viruses in A549 and Huh7 cells.** A549 or Huh7 cells were infected with WT or Δ1-642/poly(A) mutant viruses at a multiplicity of infection (MOI) of 0.1. The supernatants from virus-infected cells at different time points were collected and subjected to focus forming assay to determine the virus titers.

**Figure S4**

**Fig S4. The viral loads of different tissues in moribund WT- or Δ1-642/poly(A)-infected ICR mice at 7 dpi.** The tissues were homogenized and then used for focus forming assay to determine the viral titer.

**Figure S5**

**Fig S5. Immune protection of Δ1-642/poly(A) against intracranial (i.c.) TBEV challenge in ICR mice. (A)** Flowchart of immunization and WT virus challenge in mice. 4-week-old ICR mice were intramuscularly (i.m.) immunized with 10^6^ FFU Δ1-642/poly(A) mutant (n=5). On day 30 post-immunization, mice were challenged i.c. with 1 FFU of WT TBEV. Survival **(B)** and body weight changes **(C)** were monitored daily for 14 days after challenge. The values represent mean ±SD in each group, each symbol represents a mouse. *p< 0.05, **<0.01.

**Table S1. Sequence of primers used in the study.**

| **Primer Name** | **Genome position** | **Utilities** | **Sequence** |
| --- | --- | --- | --- |
| TBEV-Neudoerfl-9718-F | 9718 | PCR/Fusion PCR/RT-PCR | atggccaagaccaggaaggac |
| TBEV-Neudoerfl-11141-R | 11141 | RT-PCR | agcgggtgtttttccgagtcac |
| TBEV-Neudoerfl-R | 11027 | PCR | AAAAAAAAAAAAAAAAAAAAatacaaaattccccctcgg |
| pACYC-R | __ | Fusion PCR/RT-PCR | atccttcaactcagcaaaag |
| TBEV-3'UTR-509 del-F | __ | 509 del construction | ataatctaaacccagttttgtgagaccccctgc |
| TBEV-3'UTR-509 del-R | __ | 509 del construction | gggtctcacaaaactgggtttagattattgag |
| TBEV-3'UTR-592 del-F | __ | 592 del construction | atctaaacccagaaggagggaagagagaaattg |
| TBEV-3'UTR-592 del-R | __ | 592 del construction | tctcttccctccttctgggtttagattattgag |
| TBEV-3'UTR-642 del-F | __ | 642 del construction | aatctaaacccagaatacaaaattccccctcgg |
| TBEV-3'UTR-642 del-R | __ | 642 del construction | ggggaattttgtattctgggtttagattattg |
| TBEV-forward | 1 | RT-PCR | agattttcttgcacgtgtgtg |
| TBEV-Neudoerfl-2612 C | 2612 | RT-PCR | tcaaatgtctcctttatggctg |
| TBEV-4801 V | 4801 | RT-PCR | caccgaacggatggagctccg |
| **Primer Name** | **Genome position** | **Utilities** | **Sequence** |
| TBEV-4900 C | 4900 | RT-PCR | aggcatgaacctgcactgtctc |
| TBEV-Neu-4456 V | 4456 | RT-PCR | cacttgactgagctcgag |
| TBEV-Neu-6743 C | 6743 | RT-PCR | aagagtagcaaggaggcc |
| TBEV-6465 V | 6465 | RT-PCR | tggagatgtgttgagcggaatg |
| TBEV-8636 C | 8636 | RT-PCR | actccattgatcagtgacgccg |
| TBEV-8256 V | 8256 | RT-PCR | attccaactgcagtggggcgg |
| TBEV-Neu-9869 C | 9896 | RT-PCR | agttcatcttggtctcggc |
